# Rare concurrent ocular myasthenia gravis and Graves’ ophthalmopathy in a man with Poland syndrome: a case report

**DOI:** 10.1186/s12883-020-02022-6

**Published:** 2020-12-10

**Authors:** Jingqun Tang, Chao Qin

**Affiliations:** Department of Neurology, the First Affiliated of Guangxi Medial University, Guangxi Zhuang Autonomous Region, Nanning, 530021 China

**Keywords:** Ocular myasthenia gravis, Graves’ ophthalmopathy, Poland syndrome, Ptosis, Exophthalmos, Malformation, Case report

## Abstract

**Background:**

Ocular myasthenia gravis and Graves’ ophthalmopathy are autoimmune diseases that are mediated by membrane receptors and share many identical clinical processes. Poland syndrome is a rare congenital deformity characterized by defects of the ipsilateral hand and the chest wall, and it is usually associated with hypoplasia of ipsilateral pectoral muscles and homolateral breast. However, to the best of our knowledge, the co-occurrence of these diseases has never been reported. In this study, we present a man with Poland syndrome who was diagnosed with Graves’ ophthalmopathy and ocular myasthenia gravis in succession.

**Case presentation:**

A 43-year-old man presented with bilateral upper eyelid ptosis, bilateral eye protrusion, bilateral eye movement disorder and malformation of the right hand. Asymmetrical malformation of the chest wall and ipsilateral hand deformity were shown as Poland syndrome. He was diagnosed with ocular myasthenia gravis and Graves’ ophthalmopathy on the basis of clinical manifestations and laboratory examinations, including bilateral exophthalmos and progressive asymmetrical ophthalmoparesis without pupillary dysfunction, positive autoantibody tests, repetitive nerve stimulation tests, and computed tomography scans. Treatments with pyridostigmine bromide, thymectomy, and prednisone led to partial clinical improvement. After 13 months of follow-up, the symptoms of drooping eyelids were partially improved, but the eyeball protrusion and right hand deformity remained unchanged.

**Conclusions:**

We report the first case of co-occurrence of ocular myasthenia gravis, Graves’ ophthalmopathy, and Poland syndrome. Genetic predisposition and immune dysregulation might be the pathogenesis of the association.

## Background

Graves’ ophthalmopathy is a common endocrine organ-specific autoimmune thyroid disease associated with a combination of genetic, environmental, and immunological factors. Ocular myasthenia gravis is an acquired autoimmune disease mediated by autoantibodies targeting postsynaptic proteins at the neuromuscular junction (NMJ), leading to neuromuscular transmission disorders and consequent fatigable weakness. Ocular myasthenia gravis shares many clinical characteristics with Graves’ ophthalmopathy, including organ specificity with a clear pathogenic effect of antibodies, pathological mechanisms, such as dysregulation of the immune system, and the implication of genetic predisposition. Poland syndrome is a rare inborn deformity characterized by a congenital defect of the pectoralis major and various ipsilateral upper extremity anomalies and homolateral breast hypoplasia. A combination of these diseases is highly rare, and to the best of our knowledge, no published description has reported such a combination previous. In this study, we describe the case of a middle-aged male who presented with innate right hand and chest wall deformities and acquired ocular symptoms and was subsequently diagnosed with concurrent Graves’ ophthalmopathy, Poland syndrome, and ocular myasthenia gravis.

## Case presentation

A 43-year-old man, an outpatient, presented with a history of bilateral upper eyelid ptosis alternately for 5 years. He described symptom fluctuation as worse during the afternoons and evenings but better the next day after a good night’s sleep without any treatment. He reported no limb muscle weakness, hoarse voice, difficulty breathing, chewing weakness, dysphagia, or headaches. Fourteen months ago, he suffered from hyperhidrosis and irritability. Simultaneously, he noted bilateral eye protrusion, limitation to abduct his right eye, and intermittent horizontal diplopia. Two months later, he started experiencing limitation to adduct his left eye. These new symptoms led him to seek an assessment from an endocrinologist, and he was clinically diagnosed with Graves’ disease with hyperthyroid-associated ophthalmopathy (also known as Graves’ ophthalmopathy). Thyroid function tests showing elevated free thyroxine (T) 3, elevated free T4, depression of thyroid stimulating hormone (TSH), and positive thyroid peroxidase antibodies (TPO-Ab) and TSH receptor antibodies (TR-Ab) confirmed the diagnosis. Since then, treatment with propylthiouracil (oral dose of 0.3 g/day, 0.15 g each time, twice a day) was started regularly. However, no significant change in the symptoms of ptosis and eyeball movement disorders was observed after treatment for more than 1 year.

**Fig. 1:**
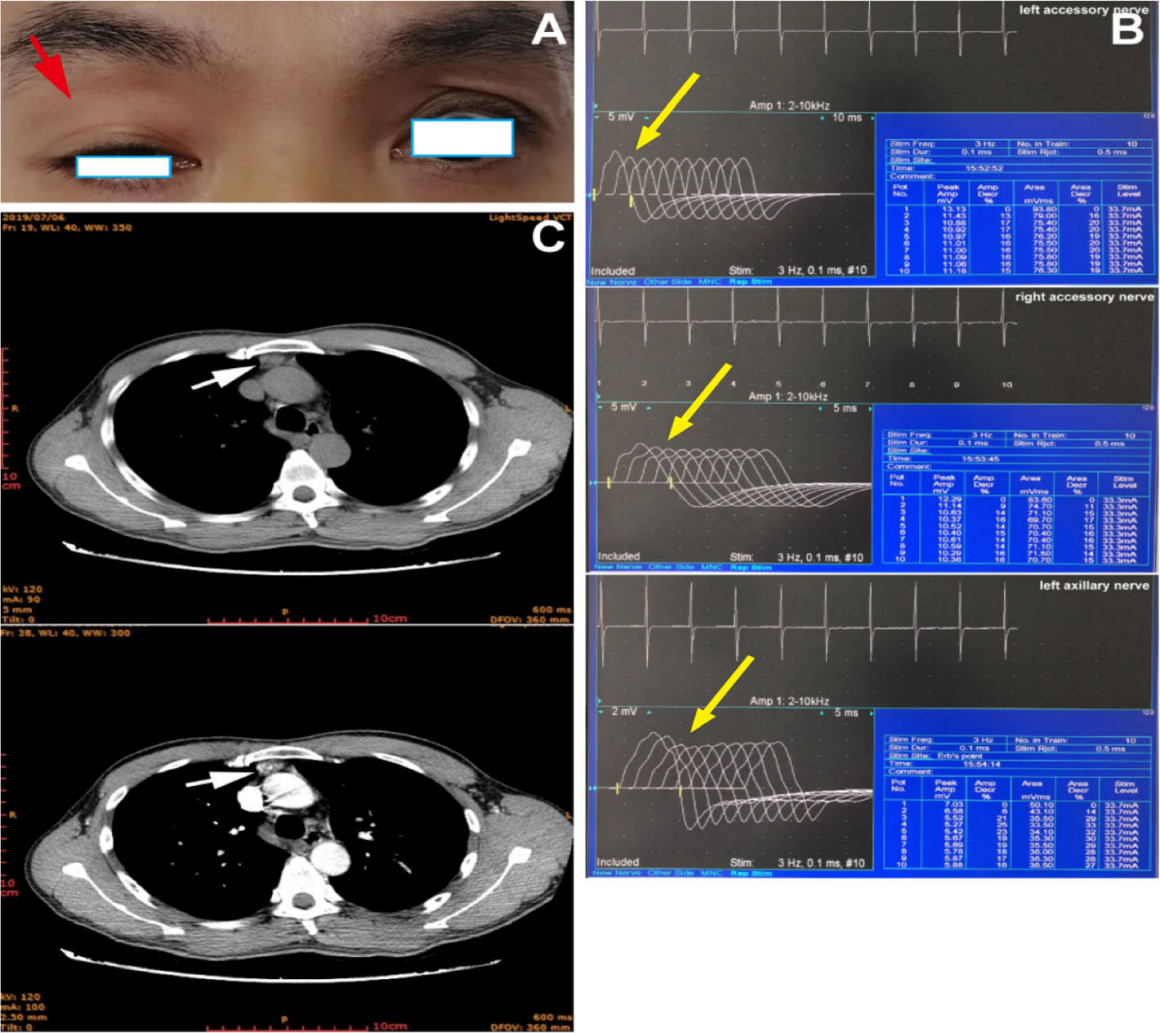
Manifestations and auxiliary examinations of myasthenia gravis. **a** Ptosis of the right upper eyelid (red arrow); **b** repetitive nerve stimulation test results of the bilateral accessory nerve and left axillary nerve were positive (yellow arrows); **c** computed tomography images showed thymic hyperplasia (white arrows)

**Fig. 2:**
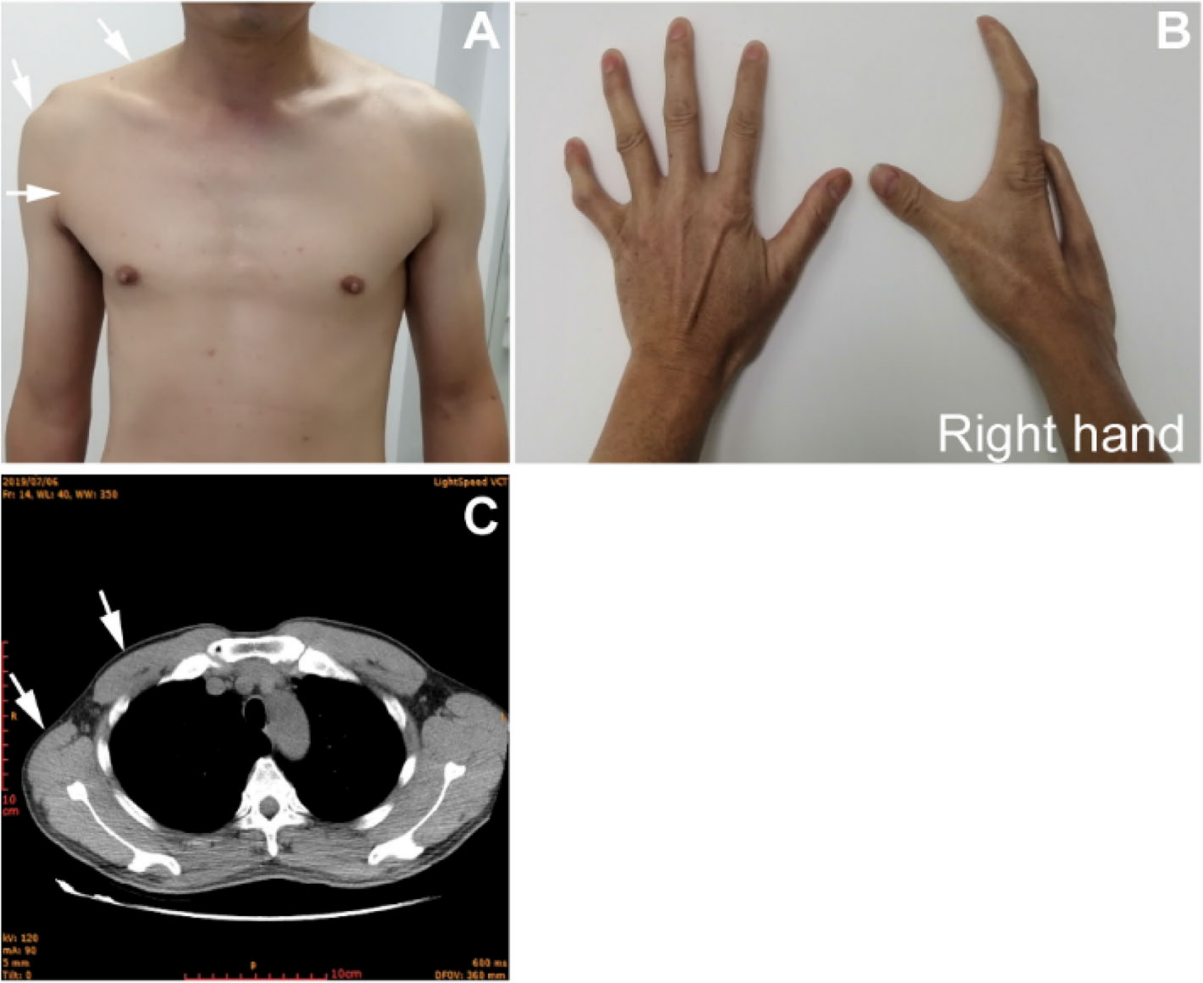
Dysplasia of the chest wall, trapezius, and hand. **a** White arrows show hypoplasia of the right pectoralis major, trapezius, and deltoid; **b** deformity of the right hand; **c** computed tomography demonstrates an incomplete defect of the right thoracodorsal muscle (white arrows)

Subsequently, a diagnosis of ocular myasthenia gravis was given based on fatigability and asymmetrical ophthalmoparesis without pupillary dysfunction by a neurological assessment. Physical examination was remarkable for ptosis of the right upper eyelid that covered the upper half of the pupil, left eyeball exophthalmos, and limitation of bilateral eyeball movement, without any pupillary dysfunction or bulbar involvement (Fig. 1a). The fatigue test of the right upper levator muscle was positive. Positivity for the neostigmine test and the repetitive nerve stimulation test (Fig. 1b) confirmed the clinical diagnosis. Acetylcholine receptor (AChR) antibody (AChR-Ab) was positive (6.273 nmol/L). Computed tomography (CT) images suggested thymic hyperplasia (Fig. 1c), and pathological examination confirmed it. Meanwhile, hypoplasia of the right thoracodorsal and deltoid muscle (Fig. 2a) and malformation of ipsilateral hands (Fig. 2b) were detected upon physical inspection. A CT scan (Fig. 2c) demonstrated hypoplasia of the right muscles. Treatment including pyridostigmine bromide (oral dose of 180 mg per day, 60 mg each time, three times a day), thymectomy, and prednisone led to partial clinical improvement. The oral prednisone treatment plan that we adopted was an escalation schedule. The first week was 10 mg per day. When no obvious worsening reaction was observed, 20 mg per day was used for the second week. When no obvious worsening reaction occurred, 60 mg per day was started from the third week. After maintaining the condition for 3 months, when the condition improved significantly, the dose was gradually reduced by 10 mg every week until the drug was stopped. After 13 months of follow-up, the symptoms of drooping eyelids were partially improved, but the eyeball protrusion and right hand deformity remained unchanged.

## Discussion and conclusion

Myasthenia gravis and Graves’ ophthalmopathy are autoimmune diseases mediated by autoantibodies targeting membrane receptors. In our case, the patient’s ptosis fluctuated initially. Next, bilateral eye protrusion, limitation of eyeball movement, and laboratory assessments of increased concentrations of thyroid hormones and thyroid-associated antibodies strongly suggested clinical Graves’ ophthalmopathy. Meanwhile, the dramatic effect of the neostigmine injection and the high level of AChR-Ab led to a second diagnosis of associated ocular myasthenia gravis.

The possible association of neuromuscular disease with thyroid disorders was first described in 1953. In the subsequent decades, this link has frequently been reported [1]. Graves’ disease occurs in approximately 5–8% of patients with myasthenia gravis [2]. The etiology of the co-occurrence of myasthenia gravis and Graves’ ophthalmopathy has not been elucidated to date. The association between these diseases may be attributable to autoimmune mechanisms. The TNF-α-863 polymorphism might be linked with ocular myasthenia gravis associated with Graves’ ophthalmopathy, indicating a molecular basis for early diagnosis [3]. In addition, human leukocyte antigen-DQ3 may play a pathogenic role in the concomitant development of myasthenia gravis and Graves’ disease [3].

Our patient presented with symptoms of ocular myasthenia gravis and Graves’ ophthalmopathy. This scenario was challenging, especially for deciding the primary or secondary etiology as well as for treatment management. Furthermore, muscle weakness might be the visible symptom of thyrotoxicosis or myasthenia gravis. Therefore, distinguishing between myasthenic and thyrotoxic neuromuscular clinical features might be useful.

Notably, the patient was found simultaneously with Poland syndrome, which was discovered by Alfred Poland in 1841. The etiology of Poland syndrome remains to be definitively established. The most widely suggested theory is an interruption of blood supply to the subclavian and vertebral arteries during the sixth week of embryonic development. Treatment is primarily characterized by an individual surgery depending on the severity of the malformation, the resulting anatomical dysfunction, and personal physical and mental aspects.

Poland syndrome, thymic hyperplasia, and Graves’ disease are often surgically treated. A see-saw relationship exists between myasthenia gravis and Graves’ disease. Treating one pathology may worsen the other pathology. Therefore, physicians should always treat MG as a difference in patients with hyperthyroidism who have new symptoms of fatigue, respiratory failure or neuromuscular weakness [4]. Conditions such as operation and infection and such medicines as anesthetics, benzodiazepines, or even antibiotics may induce a myasthenia crisis. Meanwhile, patients with myasthenia gravis may experience symptom worsening following glucocorticoid treatment initiation [5]. High-dose daily or alternate-day prednisone is more frequently associated with exacerbation than low-dose treatment, but most exacerbations are of mild to moderate severity. Other factors associated with an increased risk of initial attacks include older age, generalized myasthenia gravis, bulbar symptoms, disease severity, presence of thymoma, and thymectomy [6]. In addition, myasthenia gravis might worsen after antithyroid treatment, probably via its immunomodulatory properties, rather than its antithyroid function [7]. However, the effect of gradual escalation therapy with small doses of corticosteroids on ocular myasthenia gravis is relatively reliable and certain. Ocular myasthenia gravis and Graves’ ophthalmopathy should be treated as soon as possible to obtain a good therapeutic effect.

Myasthenia gravis associated with Poland syndrome has been presented recently [8, 9]. However, the relationship between autoimmune diseases and Poland syndrome is controversial, and a possible factor influencing pulmonary pathology has been suggested. In addition, several patients with familial Poland syndrome demonstrate autosomal dominant inheritance with incomplete penetrance [10]. Research has recently described two monozygotic twins with Poland syndrome who carried a de novo deletion at chromosome 11q12.3 involving the genes HRASLS5, RARRES3, HRASLS2, and PLA2G16, which encode proteins regulating cellular growth, differentiation, and apoptosis through Ras-mediated signaling pathways [11]. However, another study reported the presence of Poland syndrome in only one of two monozygotic twins, illustrating the complex genetic mechanisms underlying this syndrome [12]. The patient in the present study demonstrates that neuromuscular system disorders associated with inborn deformities may have a complex pathophysiological and genetic background. Although highly rare, such a combination of pathophysiological pathways may point to some common mechanisms of genetic predisposition and immune regulatory dysfunctions. The hypothesized association between these clinical entities could be proven through the records and analysis of more cases. Longitudinal epidemiological studies may also be useful and in necessary.

## Data Availability

The datasets used or analyzed during the current study are available from the corresponding author on reasonable request.

## Abbreviations

NMJ: Neuromuscular junction
TSH: Thyroid stimulating hormone
TPO-Ab: Thyroid peroxidase antibodies
TR-Ab: TSH receptor antibodies
AChR: Acetylcholine receptor
AChR-Ab: Acetylcholine receptor antibody
